# NGOs, austerity, and universal health coverage in Mozambique

**DOI:** 10.1186/s12992-019-0520-8

**Published:** 2019-11-28

**Authors:** James Pfeiffer, Rachel R. Chapman

**Affiliations:** 1 Department of Global Health, Department of Anthropology, University of Washington, Box 357965, Seattle, WA 98195-7965 USA; 2 Department of Anthropology, University of Washington, Box 353100, Seattle, WA 98195-3100 USA

**Keywords:** Mozambique, IMF, NGOs, HIV/AIDS, Universal health coverage

## Abstract

In many African countries, hundreds of health-related NGOs are fed by a chaotic tangle of donor funding streams. The case of Mozambique illustrates how this NGO model impedes Universal Health Coverage. In the 1990s, NGOs multiplied across post-war Mozambique: the country’s structural adjustment program constrained public and foreign aid expenditures on the public health system, while donors favored private contractors and NGOs. In the 2000s, funding for HIV/AIDS and other vertical aid from many donors increased dramatically. In 2004, the United States introduced PEPFAR in Mozambique at nearly 500 million USD per year, roughly equivalent to the entire budget of the Ministry of Health. To be sure, PEPFAR funding has helped thousands access antiretroviral treatment, but over 90% of resources flow “off-budget” to NGO “implementing partners,” with little left for the public health system. After a decade of this major donor funding to NGOs, public sector health system coverage had barely changed. In 2014, the workforce/ population ratio was still among the five worst in the world at 71/10000; the health facility/per capita ratio worsened since 2009 to only 1 per 16,795. Achieving UHC will require rejection of austerity constraints on public sector health systems, and rechanneling of aid to public systems building rather than to NGOs.

## Background

“There are no Ministries of Finance in Africa, there is just the IMF.” This was the startling response offered recently by Dr. Ivo Garrido, former Mozambique Minister of Health, to a question about how to increase ministry of finance support for the funding of national health systems. Dr. Garrido, who is also former President of the World Health Assembly, had been invited to the University of Washington in Seattle to give a public lecture in commemoration of the 40th anniversary of the 1978 Alma Ata Primary Health Care conference. He had been Health Minister during the first critical years of HIV/AIDS care and treatment expansion from 2005 to 2010, which saw a quadrupling of aid to the health sector, mostly from PEPFAR and the Global Fund to Fight AIDS, tuberculosis, and malaria. He recounted his experience in trying to push for increased state budget support to strengthen and expand Mozambique’s National Health Service, especially its workforce, where HIV/AIDS care and treatment was being scaled up. At closed-door sessions with the Ministry of Finance, Dr. Garrido’s requests were usually rejected. The greatest share of the new aid funding was directed to American NGOs and contractors rather than the National Health Service itself, partly because of IMF austerity conditionalities that placed caps on public sector budgets.

While hundreds of millions of new aid dollars were pouring into heavily indebted Mozambique, these IMF conditionalities—initiated under structural adjustment programs in the 1980s and then extended through the 2000s with the re-branded “Poverty Reduction Strategy” approach—ensured that the state health budget remained anemic, even in the midst of the deepening AIDS crisis [1]. The macroeconomic strategy later designed for Mozambique through the IMF’s Poverty Reduction Strategy Paper (PRSP) process included budget envelopes and ceilings that constrained state spending and prevented significant growth of “on-budget” health services investment.

Mozambique is not alone in this experience. Most African countries have implemented structural adjustment programs, and later PRSPs, in some form since the 1980s [2]. Nor is Dr. Garrido alone in his lament [2–5]. IMF conditionalities and austerity programs have crippled the ability of countries across Africa to strengthen public-sector national health services and have impeded health coverage expansion. The imposition of IMF conditionalities has been paralleled by a remarkable proliferation and expansion of international NGOs in the development and global health arenas, recruited to fill the gaps left by debilitated state services [6]. In many African countries, hundreds of health-related NGOs are fed by a chaotic tangle of multiple donor funding streams and project targets. While donors have various motivations for funding NGOs, the IMF-imposed limitations on state budget growth have been a major factor in the re-channeling of new foreign aid for health away from public services to NGOs, as well as to contractors and for-profit companies [5, 7, 8].

The new emphasis on Universal Health Coverage (UHC) in the UN Sustainable Development Goals (SDGs) raises the stakes and underscores the threat to progress in global health by the IMF’s influence and imposition of what some call “permanent austerity” [9]. The Mozambique experience provides a case study in how the NGO-driven aid model, promoted in tandem with continued austerity in the public sector, undermines progress toward achieving UHC and the SDG goals.

## Main text

### Selective primary health care and austerity

A troubling 2018 piece, commissioned by the *Lancet* for the WHO-sponsored Astana conference on primary health care to commemorate the 40th anniversary of the Alma Ata Declaration on Primary Health Care, emphasizes the key role of ministries of finance in supporting state health budgets [10]. The co-authors, who include prominent health economists who helped draft the pivotal 1993 World Bank treatise “Investing in Health,” argue in favor of health spending that targets specific vertical interventions [11]. Their line of reasoning is redolent of the “Investing in Health” argument made for a selective primary healthcare approach a generation ago, which undermined the 1978 Alma Ata Declaration’s powerful call for comprehensive Primary Health Care (PHC) and “Health for All”. Selective PHC focused on narrowly construed, targeted, “cost effective” and “feasible” interventions to address specific health issues such as under-five child mortality through, for example, immunization and oral rehydration solutions, rather than providing comprehensive health services for all and supporting intersectoral approaches to improving health [12]. In fact, some passages in the 2018 *Lancet* article almost paraphrase the infamous 1979 Walsh and Warren piece [12] that kicked off the selective PHC push when structural adjustment and austerity were beginning to be rolled out in the developing world during the 1980s debt crisis. They write, “We do not advocate for increased public expenditure on health overall, but rather for increased spending, through national UHC systems, on *specific interventions that provide good value for money and improve health equity*. This distinction is crucial to reassuring ministries of finance that additional resources will be spent well and will lead to substantial economic returns. But what sorts of resources might be required to finance this disciplined approach to UHC?” [emphasis added] [10].

This argument for more vertical programming that excludes “increased public spending for health overall” should alarm those working toward UHC. The focus on “reassuring ministries of finance”, most likely with IMF oversight in many countries, suggests that austerity will be reinforced. While this is not specifically stated in the *Lancet* piece, the vertical approach proposed there, combined with rigid constraints on public spending, will continue to invite NGOs to play outsized roles in implementing selective health interventions in ways that often undermine comprehensive public systems. Historically, vertical funding for health issues ranging from TB and malaria to immunization has been channeled through both NGOs and national health services. However, even when directed through public systems, the funding often stays off government budgets and focuses on disease-specific materials and activities rather than health system fundamentals such as workforce and infrastructure. Given IMF caps on public spending, vertical funding is often undertaken by NGOs or other external agencies, such as Gavi or foreign universities, who may manage the funding and work even if it is conducted within the public system itself [5]. To be sure, some of the vertical funding proposed in the *Lancet* article might continue to flow through national health services as well as NGOs—this is not specified—but “on-budget” funding to build out national health services for comprehensive health care for all is clearly not what they envision. The roots of this “disciplined” approach (an insidious euphemism that actually means the constraining of public financing for public systems) extend back to the 1980s and structural adjustment, but were perhaps best articulated in the 1993 “Investing in Health” treatise itself. The Bank presented its recipe for privatization in this way: “[E]ncourage greater diversity and competition in the provision of health services by decentralizing government services, promoting competitive procurement practices, fostering greater involvement by nongovernmental and other private organizations, and regulating insurance markets” [11].

### Structural adjustment

Mozambique’s experience with the NGO model in the midst of PRSP constraints on public spending reveals the urgency for course correction to achieve UHC in Africa. After gaining independence from Portugal in 1975, the new government planned and built its National Health Service premised on the primary healthcare concept. It eventually created a system with over a thousand health units strategically located to maximize equitable access across its population [12]. However, in the late 1970s the Rhodesian government organized and trained an insurgency known as the Mozambique National Resistance (MNR, or RENAMO using the Portuguese acronym). After the fall of white minority rule in Rhodesia and the transition to Zimbabwe, support for RENAMO shifted to apartheid South Africa, and the war of destabilization raged until 1992. Rural health units and health workers were targeted, but the system managed to survive [13]. The war also drove the new government deeply into debt; in 1987, it signed onto a structural adjustment program that quickly cut the national health budget. Part of the package was an invitation to international NGOs to set up shop in Mozambique.

By the early 1990s, NGOs had multiplied across post-war Mozambique, as the country’s adjustment program constrained both public and foreign aid expenditures on the public health system while donors favored private NGOs [14]. In the early 2000s, HIV/AIDS and other vertical aid funding from a range of donors increased dramatically. Under IMF structural adjustment—now re-branded globally as “Poverty Reduction Strategies (PRSPs)”—constraints on investment in the public National Health Service continued. In Mozambique the PRSP was known as by its Portuguese acronym PARPA (*Plano de Ação para a Redução da Pobreza Absoluta,* or Action Plan for the Reduction of Absolute Poverty*).* During this same period, Western European bilaterals and the Mozambique Ministry of Health created a “common fund” using the SWAp (Sector Wide Approach to planning) approach that allowed donors to contribute to a common fund managed jointly with the Ministry of Health to support system strengthening [15]. While many applauded the fund as a way for Western donors to support health-system fundamentals, it has always represented a small fraction of total aid to the health sector, varying from 5 to 20% [15].

### HIV and the “golden age” of global health assistance

In 2004, the USA introduced PEPFAR in Mozambique at eventually half a billion dollars per year, roughly equivalent to the entire budget of Mozambique’s Ministry of Health. To be sure, PEPFAR funding has helped put thousands on antiretroviral treatment and must be seen as a public health success. But over 90% of PEPFAR resources flow “off-budget” to United States NGO “implementing partners,” with little left for the health system itself. These partners normally include many of the large US-based organizations, such as Abt Associates, FHI360, JSI, and EGPAF [16]. Little if any of this funding was channeled through the common fund.

Some observers, such as Shiffman et al., initially hoped that PEPFAR funding would “raise all health funding boats” [17]. In Mozambique, PEPFAR partners are normally allocated to specific provinces around the country where, in principle, they are to collaborate with local health-system authorities to support the scale-up of HIV care and treatment services [16]. Nearly all HIV-positive patients receive their care through the National Health Service; PEPFAR partners generally do not set up separate clinics, instead grafting their activities onto existing facilities. Their support normally includes health workforce training (usually brief two to three-day seminars), some HIV-specific materials, technical assistance, data collection, and support for HIV-specific community health workers, with subcontracting to local NGOs for social support and adherence. All of this funding is spent separately from the state budget. “Rather than raising all health funding boats,” little if any PEPFAR funding has supported basic health-system building blocks such as transport, infrastructure, or health workforce expansion, and health-system support from other donors has not increased [16]. Frontline doctors and nurses are often tasked with ever more HIV-related work in increasingly overcrowded facilities [18].

PEPFAR also requires that its partners collect huge amounts of data that are eventually fed into a global database managed by PEPFAR. Rather than using PEPFAR funds to strengthen Mozambique’s health information system, each NGO normally sets up its own parallel data collection system, using separate forms, data entry, data transfer systems, and analysis. Health-worker time is a zero-sum game, so facility-service providers are frequently diverted to support data collection for external partners (on similar dynamics in Tanzania, see Sullivan [19]).

The scale of NGO proliferation has also led to an internal brain-drain crisis, as public-sector health workers are lured out of the system to work for NGOs at much higher salaries. A study from 2012 quantified recent loss of Mozambican physicians to international NGOs and agencies, and found that nearly half of those who left had joined PEPFAR-funded agencies [20]. They moved from providing clinical care or managing service delivery, to occupying desk jobs in NGO offices. Leadership in the Ministry of Health has become increasingly frustrated with the lack of transparency and coordination among donors and NGOs, and the internal brain drain they are causing. In 2017 alone, the National Health Service reportedly lost nearly 300 health workers to higher-paying jobs with NGOs [21]. Thus, not only are health facilities burdened with ever-new layers of work from NGO vertical projects, they are also losing their own staff to manage the burden. In some high-volume health facilities where we have conducted implementation research, we recorded a seven-hour average waiting time among pregnant women for a seven -minute antenatal consultation [18].

PEPFAR implementing partners budgets are opaque [22]. Internal NGO budgets are not public, nor are they shared with the Ministry of Health; therefore rigorous costing analyses are rare or nonexistent. Moreover, many of these partners are known for their high administrative costs, very high six-figure expatriate salaries with major benefits, comparatively lavish in-country offices, big fleets of new SUVs, and large staffs dedicated to managing subcontracts, collecting data, and managing operations—while the health systems they are ostensibly meant to support remain dilapidated and understaffed.

The massive effort led by PEPFAR to bring antiretroviral (ARV) treatment to Africa and Mozambique has had tremendous success by many measures, which must be recognized and supported. In Mozambique, by 2016 an estimated 86% of HIV-positive pregnant women had received ARVs [23]. Over 54% of the HIV-positive population has started antiretroviral therapy (ART). But that also means that 46% (nearly a million people) still need to access treatment; it is estimated that 40% of those who are infected have not been tested and do not know their status. An adherence crisis looms, as new data indicate high loss-to-follow-up (poor retention in care once treatment is started, leading to poor adherence) across all groups [24]. Achieving the current 90–90–90 goal (90% tested, 90% of HIV positives started on ART, and 90% of those on treatment with viral load suppression) [24] will be beyond the reach of Mozambique and other African countries unless health system capacity itself improves dramatically, meaning major workforce and infrastructure expansion. If the huge increase in aid funding for HIV over the last 15 years had been invested in the National Health Service itself, including its HIV care and treatment capacity, the 90–90–90 targets would not be so far off.

These dynamics are not restricted to PEPFAR and HIV/AIDS programs. In Mozambique, as in many other African countries, a range of donors finance vertical projects that focus on everything from TB, malaria, reproductive health, and immunization to mental health and NCDs. These project are managed by NGOs and are frequently grafted onto the health system, without the provision of systemic support to increase workforce and infrastructure. There are also vertical projects that flow through the health system. For example Gavi, the Vaccine Alliance, a global public–private partnership to support immunization services, normally sets up separate accounts to support the health system itself around the specific needs related to immunization coverage, although some Gavi funding also flows to NGOs [25]. Some malaria bed-net distribution projects flow entirely through NGOs [26]. In several large health facilities in central Mozambique, where we recently conducted HIV-related research, there were as many as four different NGO projects, each loading its own disease-specific community health workers (and data collection processes) onto understaffed health centers. This created a confusing tangle of interests and objectives that land on desperately overworked nurses [27]. In one facility, NGO-supported community health workers (known as *activistas*) were financed by three different organizations to support HIV treatment follow-up, community health organizations, and maternal child health (MCH). Often, MCH nurses did not know which *activistas* were working for whom. Other NGOs supported pediatric HIV activities and laboratory work at the same facilities.

### Impact on universal health coverage

What has this model done for actual population coverage of the health system in Mozambique? Surely the quadrupling of aid funding must have led to improved access to health services across the country? Unfortunately, the data tell a different story. After nearly 15 years of major increased foreign aid for health from donors that have included PEPFAR, Global Fund, UNICEF, USAID and other bilaterals to NGOs, health system coverage has barely changed, as shown below. Despite the many challenges, the Mozambique system has achieved remarkable progress and managed to continue functioning and provide care to millions. However, while there has been progress in the fight against HIV/AIDS, as of 2014 the workforce per population ratio was still among the five worst in the world at 71/10000; the health facility/per capita ratio had actually worsened since 2009 to only one per 16,795 by 2015 [28]. Furthermore, as a result of PRSP austerity constraints, the proportion of overall government expenditure declined from 13.4% in 2006 to 11.9% in 2009, 7.8% in 2014, and 9% in 2015, moving away from the Abuja target of 15% [29–31]. In 2011, 65% of the population still lived more than 45-minute walking distance from the closest health facility. Many health facilities still lack basic infrastructure for quality health care; almost 50% of health centers do not have access to electricity and 60% still lack access to clean water [32]. Even with the massive infusion of new vertical funding, progress toward achieving Universal Health Coverage as these data show has been minimal, and on some measures Mozambique has lost ground.

## Conclusions

Promotion of Universal Health Coverage through the SDGs has stimulated important and lively debates about what “coverage” means, which services should be included, what is the best public-private balance, how should it be financed, and a range of other concerns. In heavily indebted poor countries such as Mozambique, however, the challenges are bigger than those found in wealthier parts of the world, but the choices are simpler. The great majority of Mozambicans who can access care will continue to get their care through the public National Health Service. To expand health coverage, the health service itself will need to expand, to be able to provide its comprehensive primary health care package to all.

One major lesson from the AIDS treatment scale-up effort in Africa is that treatment coverage and retention can be attained only through a strong comprehensive primary health care system. HIV-positive people may present at health facilities with a range of other conditions that might include malnutrition, malaria, TB, mental illness or dysentery, to name only a few. They may come with children in tow with their own health issues, and perhaps have moved multiple times, so they need comprehensive records that can follow them. At the beginning of the ART scale-up in Mozambique, the Ministry of Health quickly realized that HIV treatment services needed to be integrated into the existing primary health care facilities in order to reach coverage targets, ensure equity, and link to other services that patients needed. The preliminary free-standing HIV-centric and vertical “day hospital” approach was quickly abandoned [33]. The same of course must be said of TB, malaria, immunization, or newly emerging chronic illnesses programs of all types. The idea that UHC can be achieved in some form through selective vertical disease-specific approaches identified and chosen through “cost-effectiveness” modelling is as dangerously ill-conceived now as it was in 1979 when selective primary health care (PHC) was first proposed. If the donor world chooses the path of selective disease-specific support it is likely to decide, once again, to finance NGOs rather than health systems, in order to reach specific, narrow targets.

With the world now focusing its attention on UHC, the urgent need to build and expand public-sector health services should stand out in high relief. The Mozambique experience provides an important red flag alerting us to the dangers of the NGO vertical model promoted in tandem with continued IMF-imposed austerity on public spending. The Mozambique case is not unique: it is typical of poor countries throughout sub-Saharan Africa [3, 5]. UHC can become a focal point for advocacy and activism to demand greater public spending in poor countries, renew calls for debt forgiveness, and insist that foreign aid be directed toward the long-term strengthening and expansion of national health services rather than further support for a chaotic and inefficient NGO patchwork. The occasion of the 40th anniversary of the Alma Ata Health for All declaration of 1978 offered an opportunity to revisit the values and vision of that agreement, which can still provide a template for the new drive for UHC.

## Data Availability

Not applicable

## Abbreviations

ART: Anti-Retroviral Therapy
NGO: Non-Governmental Organization
PEPFAR: President’s Emergency Program for AIDS Relief
PRSP: Poverty Reduction Strategy Paper
SWAp: Sector-Wide Approach to planning
SDG: Sustainable Development Goals
UHC: Universal Health Coverage
